# Use of Tiger Nut (*Cyperus esculentus* L.) Oil Emulsion as Animal Fat Replacement in Beef Burgers

**DOI:** 10.3390/foods9010044

**Published:** 2020-01-03

**Authors:** Julliane Carvalho Barros, Paulo E. S. Munekata, Francisco Allan Leandro de Carvalho, Mirian Pateiro, Francisco J. Barba, Rubén Domínguez, Marco Antonio Trindade, José Manuel Lorenzo

**Affiliations:** 1 Faculdade de Zootecnia e Engenharia de Alimentos, Universidade de São Paulo, São Paulo 13.635-900, Brazil; jullianebarros@usp.br (J.C.B.); francisco.allan@usp.br (F.A.L.d.C.); trindadema@usp.br (M.A.T.); 2 Centro Tecnolóxico da Carne de Galicia, 32900 Ourense, Spain; paulosichetti@ceteca.net (P.E.S.M.); mirianpateiro@ceteca.net (M.P.); rubendominguez@ceteca.net (R.D.); 3 Food Science, Toxicology and Forensic Medicine Department, Faculty of Pharmacy, Universitat de València, Nutrition and Food Science Area, Preventive Medicine and Public Health, s/n 46100 Burjassot, València, Spain

**Keywords:** fat-replacer, fatty acid profile, physicochemical characterization, sensory acceptance

## Abstract

The present study evaluated the replacement of beef fat in beef burgers using a tiger nut (*Cyperus esculentus* L.) oil emulsion, in order to reduce total fat and saturated fatty acids in the studied samples. Three formulations were processed: Control—100% beef fat; tiger nut 50% (TN50)—50% of beef fat replaced using tiger nut oil emulsion and tiger nut 100% (TN100)—100% of beef fat replaced by tiger nut oil emulsion. The physicochemical parameters were affected after fat replacement. Moreover, the protein and fat contents decreased in those sample with tiger nut oil emulsion, thus the formulation TN100 can be considered as “reduced fat content”. Regarding color, an increased L* and b* value parameters was observed after TN100 while the values of a* remained similar to the Control samples. The hardness, cohesiveness, gumminess and chewiness were similar in all formulations. The addition of tiger nut oil emulsion as a substitute for beef fat reduced saturated fat and increased the mono- and polyunsaturated fatty acids. Oleic acid was found to be in highest proportions in burgers. The TN100 samples were considered as acceptable by consumers. Therefore, total replacement of beef fat using tiger nut oil emulsions in beef burger resulted in a well-accepted and healthier meat product with reduced total and saturated fat contents, as well as increased unsaturated fatty acids.

## 1. Introduction

Beef burgers are widely consumed by a variety of consumers, being especially attractive for young people, because of their sensory characteristics and fast preparation [1,2]. However, the excessive consumption of this product can be harmful to human health. Some studies have shown that beef burger contains from 9% to 20% animal fat in formulation (the amount of fat normally used in this product), with high saturated fatty acid (SFA) content (31% to 42%) [3,4,5].

It is known that the diets rich on fat (particularly saturated fat) can increase the risk related to develop coronary heart disease, according to the World Health Organization [6]. Furthermore, this organization also stated that oils rich in oleic acid should be consumed as a contribution to the daily total intake of fat after adequate consumption of polyunsaturated fatty acids (PUFA), since the intake of oleic acid is associated with reduced risk to develop cardiovascular diseases.

For this reason, in recent years, the development of new formulations or improvement of traditional food products in order to reduce both fat and SFA contents became a major goal for the food industry, particularly in the meat sector [7,8,9,10]. Thus, due to the high fat content of burgers (with an elevated amount of SFA), the lipid modification of this product by replacing animal fat with other lipid sources has been proved to be a good strategy in order to improve nutritional quality and maintain sensory properties without changing dietary habits [11].

To this regard, the substitution (25% to 100%) of pork back fat by sunflower oil gelled emulsion in burger improved nutritional quality, since fat, cholesterol and SFA contents were reduced, while PUFA were increased [12]. The same trend was observed in burgers containing a mixture of pre-emulsified olive, corn and deodorized fish oils [13]. Additionally, besides reducing burger fat content by replacing fat with canola oil [14] and olive and linseed oils’ mixture emulsion [15], these authors observed that vegetable oils did not affect negatively the sensory characteristics of the obtained meat products.

In this line of thought, tiger nut oil (*Cyperus esculentus* L., commonly known as “chufa”) has relevant nutritional and technological characteristics for the development of healthier meat products. This weed is a member of the *Cyperaceae* family [16] that are commonly found on tropical and Mediterranean regions [17]. The oil obtained from the tubers of tiger nuts is characterized by the low SFA (<23%) and high unsaturated fatty acid (UFA) (>78%) contents, wherein oleic acid is the predominant fatty acid (>65%) [18,19,20]. Moreover, tiger nuts seeds contain around 16–25% of lipids and have an almond-like flavor. Thus, tiger nuts can be considered as an emerging resource of lipids to replace animal fat and improve the nutritional quality and shelf life of meat products [21,22,23].

With this is mind and the scarcity of scientific studies regarding the use of tiger nut oil in meat products, the present study aimed to reduce the total fat content and SFA in beef burgers. For this purpose, animal fat was partially and totally replaced by tiger nut oil emulsion in beef burgers and their proximate composition, physicochemical parameters, fatty acids profile and sensory characteristics were evaluated.

## 2. Materials and Methods

### 2.1. Preparation of Tiger Nut Oil Emulsion

The study was performed in the Centro Tecnolóxico da Carne (CTC) de Galicia (San Cibrao das Viñas, Spain). In order to prepare the tiger nut oil emulsion with Prosella (Prosella VG NF4, Colin Ingrédients, Mittelhausen, France), its elaboration was performed one day before the processing of burgers [24]. The Prosella was composed of jellifying agents (calcium sulphate and sodium alginate), wheat glucose syrup (7.4%), a stabilizer (disodium diphosphate, added P_2_O_5_: 9.58%) and an antioxidant (sodium ascorbate), which retain oils in its structure and can be used as animal fat replacer. For tiger nut oil emulsion preparation, water (56 g/100 g) and tiger nut oil (37.3 g/100 g) were mixed for 1 min in a bowl cutter (Sirman, mod C15VV, Marsango, Italy). The Prosella powder (6.7 g/100 g) was added and homogenized during 3 min and then left rest for 2 h, then the mixture was refrigerated at 4 °C until needed.

### 2.2. Beef Burgers Manufacture

Three different formulations were processed: Control—containing 100% beef fat (5 g/100 g; formulation with low amount of fat added); tiger nut 50% (TN50)—50% of beef fat (2.5 g/100 g) was replaced by tiger nut oil emulsion (2.5 g/100 g) and tiger nut 100% (TN100)—100% of beef fat was replaced by tiger nut oil emulsion (5 g/100 g). The others ingredients used in all formulations were meat (85.8 g/100 g), salt (1.2 g/100 g) and water (8 g/100 g). Prime cuts of foreshank and beef fat were used in burger processing. All visible fat and connective tissue were removed manually from the meat. For burger processing, firstly primal cuts of foreshank and beef fat and/or tiger nut oil emulsion were ground through an 8 mm and 6 mm diameter mincing plate in a refrigerated mincer machine (La Minerva, Bologna, Italy), respectively. The meat batter was mixed together with the remaining ingredients until complete homogenization and shaped in a burger format. The different treatments were shaped (10 cm diameter and 1 cm height) in a manual burger machine. A total of 2 kg of mass was prepared, resulting in 25 burgers in each treatment and weighing 80 g each. After processing, the treatments were evaluated for their proximate composition, physicochemical parameters, fatty acids profile and sensory characteristics.

### 2.3. Proximate and Physicochemical Analysis of Beef Burgers

#### 2.3.1. Proximate Composition, Carbohydrates and Energy Content

The proximate composition of the different beef burger treatments were evaluated according to International Organization for Standards (ISO), where it was determined protein [25], moisture [26] and ash [27] content. Total fat was determined according to the Approved Procedure Am 5–04, established by the American Oil Chemists’ Society [28], while total carbohydrate content was determined by a difference calculation [29]. The energy content was calculated based on 17 kJ/g for protein and carbohydrates and 37 kJ/g for fat according to European Commission [30].

#### 2.3.2. Color and pH

Color parameters (L*—brightness, a*—greenness/redness and b*—blueness/yellowness) were measured in the CIELAB space using a portable colorimeter (CR-600d, Minolta Co. Ltd., Osaka, Japan). The device was set to pulsed xenon arc lamp, 10° viewing angle geometry, and 8 mm aperture. The pH was measured in the beef burgers using a digital pH-meter (Hanna Instruments, Eibar, Spain) equipped with a penetration glass probe.

#### 2.3.3. Cooking Loss and Texture Profile Analysis

For analysis of cooking loss and instrumental texture, the burgers were cooked using vacuum package bags and introduced in a water bath with automatic temperature control (JP Selecta, Precisdg, Barcelona, Spain) until they reached an internal temperature of 70 °C, monitoring the heat by a thermocouples type K (Comark, PK23M, St Neots, UK) connected to a data logger (Comark Dilligence EVG, N3014). Cooking loss was measured by difference in weight between cooked and raw samples.

Texture profile analysis (TPA) (hardness, springiness, cohesiveness, gumminess and chewiness) was measured by compressing to 60% (cylindric probe with flat surface area of 19.85 cm^2^) and the force-time curves were recorded at 3.33 mm/s crosshead speed. The texture parameters were obtained using Texture Exponent 32 software (version 1.0.0.68, StableMicro Systems, Vienna Court, UK).

#### 2.3.4. Lipid Oxidation

For the determination of lipid oxidation, the TBARS index (2-thiobarbituric acid; secondary products of the lipid oxidation) was determined [31] at time zero to assess the degree of lipid oxidation in the different samples. Thiobarbituric acid reactive substances (TBARS) values were calculated from a standard curve of malonaldehyde (MDA) with 1,1,3,3-tetraethoxipropane (TEP) and expressed as mg MDA/kg sample.

### 2.4. Fatty Acid Analysis of Beef Burger

For fatty acid analysis, total fat was extracted following the method described by Bligh and Dyer [32] with modifications. Briefly: 10 g of sample were homogenized with 10 mL of chloroform and 20 mL of methanol for 30 seconds. After, the mixture was added of 10 mL of chloroform and 10 mL of NaCl (1% in distilled water) and homogenized for 30 seconds. Then, the chloroform layer (with the fatty acids) was separated from the residues and aqueous layer by centrifugation (4000 rpm for 10 min). Finally, chloroform was evaporated using N_2_ gas and the extracted fatty acids were reserved for transesterification.

The fatty acids were transesterified according to the procedure previously described by Domínguez et al. [33], with some modifications: for the fatty acids transesterification, twenty milligrams of extracted fat dissolved in 1 mL of toluene were mixed with 2 mL of a sodium methoxide (0.5 N) solution, vortexed during 10 s and allowed to stand for 15 min at room temperature. Then, 4 mL of a H_2_SO_4_ solution (10% of H_2_SO_4_ in methanol) was added, vortexed for a few seconds and vortexed again before adding 2 mL of saturated sodium bicarbonate solution. For the extraction of fatty acid methyl esters, 1 mL of hexane was added to the samples, vortexed for 10 s and the organic phase was then transferred to an appropriate GC vial.

Separation and quantification of FAMEs were carried out using a gas chromatograph (GC-Agilent 7890B, Agilent Technologies, Santa Clara, CA, USA) equipped with a flame ionization detector (FID) and PAL RTC-120 auto sampler. One microliter of sample was injected in split mode (1:50). The injector was maintained at 250 °C and 64.2 mL/min of total flow. For the separation of FAMEs, a DB-23 fused silica capillary column (60 m, 0.25 mm i.d., 0.25 μm film thickness; Agilent Technologies) was used. Chromatographic conditions were as follows: initial oven temperature of 50 °C (held for 1 min), first ramp at 25 °C/min to 175 °C, second ramp at 4 °C/min to 230 °C (held for 5 min) and third ramp at 4 °C/min to a final temperature of 240 °C (held for 2.75 min). Helium was used as a carrier gas at a constant flow-rate of 1.2 mL/min, with the column head pressure set at 22.9 psi. The FID detector was maintained at 280 °C, while the operational flows were set as 40 mL/min of H_2_, 450 mL/min of air and 30 mL/min of makeup flow. The total time for chromatographic analysis was 30 min. Data acquisition and equipment control was carried out using the software Mass Hunter GC/MS Acquisition B.07.05.2479 (Agilent Technologies), while the data analysis was carried out with the software Mass Hunter Quantitative Analysis B.07.01. Individual FAMEs were identified by comparing their retention times with those of authenticated standards (FAME Mix-37 components-; docosapentaenoic acid (C22:5n-3; DPA); *trans*-11 vaccenic acid (11t-C18:1; TVA); *cis*-vaccenic acid (18:1n-7, CVA, Supelco, Madrid, Spain) and conjugated linoleic acid (9c,11t-C18:2, CLA, Matreya, State College, PA, USA) and the results were expressed as g/100 g of total fatty acids identified.

The total SFA, MUFA and PUFA contents as well as the atherogenic (AI) and thrombogenic (TI) index [34] and the hypocholesterolemic/hypercholesterolemic ratio (h/H) [35] were calculated. The nutritional indices AI, TI and h/H are calculated to evaluate the nutritional quality of the fat composition of burgers. The IA and TI indices are used to express the effects of each fatty acid on cardiovascular risk and h/H based on the functional effects of fatty acids on cholesterol metabolism [33,34].

### 2.5. Sensorial Acceptance Evaluation of Beef Burgers

Sensory analysis was conducted by 68 consumers (with ages between 29 and 40 y and from both genders) from Ourense (Spain). The treatments were evaluated in raw and cooked samples, to determine whether the panelist liked or disliked of the different levels (50% and 100%) of beef fat substitution by tiger nut oil emulsion in relation to Control. Consumers evaluated the beef burgers by the acceptance test using a 7-point hedonic scale, which ranged from “1—disliked much” to “7—liked much”, for the following attributes: raw burgers—color and odor and cooked burgers—texture, flavor, fatty flavor and overall quality. In addition, consumers were asked to order the samples according to their preference and purchase intentions. The burgers were cooked in an oven (Rational Combi Master^®^ Plus CMP61, Landsberg am Lech, Germany) equipped with a core temperature probe, until they reached an internal temperature of 70 °C. The samples were cut in 2 cm^3^ portions, which were individually wrapped in foil and marked with a random 3-digit code. The samples were kept warm in a heater at 55 °C until the testing (up to 30 min). To avoid the possible effects of the order of presentation, the samples were presented to panel members in a random order [36] and served to panelist together with water and toast.

### 2.6. Statistical Analysis

Statistical analyses were performed using the Statistical Analysis System (SAS version 9.4, SAS Institute Inc., Cary, NC, USA). Normal distribution and homogeneity of variance were previously tested (Shapiro-Wilk). Data were submitted for analysis of variance (ANOVA) and Tukey test, when ANOVA had a significant effect (*p* < 0.05). For proximate composition, physicochemical analysis and fatty acids data, treatments were considered as fixed effects and replications (the whole experiment was repeated twice) as a random effect, whereas for sensory analysis consumers were additionally included in the model as a random effect (each panellist tasted three samples, one from each formulation, in a single session). The statistical evaluation for the preference test was performed using the Friedmann test, with Newell and McFarlane tables (*p* < 0.05) and the purchase intention was evaluated with the qui-squared test (*p* < 0.05).

## 3. Results

### 3.1. Proximate Composition and Physicochemical Parameters of Beef Burger

Proximate composition and physicochemical results of the beef burgers are shown in Table 1. Proximate composition was affected by total replacement of beef fat by tiger nut oil emulsion. Replacing 100% of beef fat by tiger nut oil (TN100 treatment) decreased protein (*p* < 0.001) (18.75 g/100 g in TN100 samples vs. ≈19.15 g/100 g in the other two batches) and fat content (*p* < 0.001) (3.01 g/100 g in TN100 samples vs. ≈4.38 g/100 g in the other two batches). Thus, an approximately 32% reduction in fat content was observed in the TN100 treatment. In a similar way, the carbohydrates content also decreased in the TN100 (0.58 g/100 g) compared to TN50 (1.20 g/100 g) and Control (1.09 g/100 g) samples. In contrast, the highest values of moisture were obtained in TN100 (*p* < 0.001) and the ash content was higher in TN50 and TN100 samples (*p* < 0.01) than Control samples.

The differences in the proximate composition cause that the energy content showed significant differences between batches (*p* < 0.001). It can be seen that burgers with 100% of beef fat replaced by tiger nut oil (TN100 formulation) showed a reduction of approximately 14% in energy content.

The mean pH values of the different beef burger treatments ranged from 5.48 to 5.76 (*p* < 0.001; Table 1). The color parameters increased gradually as beef fat was replaced by tiger nut oil emulsion. In fact, L* values increased from 44.13 in Control to 46.94 in TN100 and b* increased from 19.19 in Control to 20.88 in TN100. In a similar way, besides a* values did not show significant differences, the values also increased from 20.86 in Control to 21.83 in TN100. In all cases, TN50 presented intermediate values.

Cooking loss decreases (*p* < 0.001) with 100% replacement of beef fat by tiger nut oil emulsion (13.27% in TN100 vs. ≈23% Control and TN50 treatments). Despite the differences found in the proximate composition and cooking loss, texture parameters were not affected by the animal fat replacement. For mean TPA values, only the springiness parameter was affected (*p* < 0.001) by total replacement of beef fat by tiger nut oil emulsion (TN100 treatment). The hardness, cohesiveness, gumminess and chewiness were similar in all formulations.

**Figure 1:**
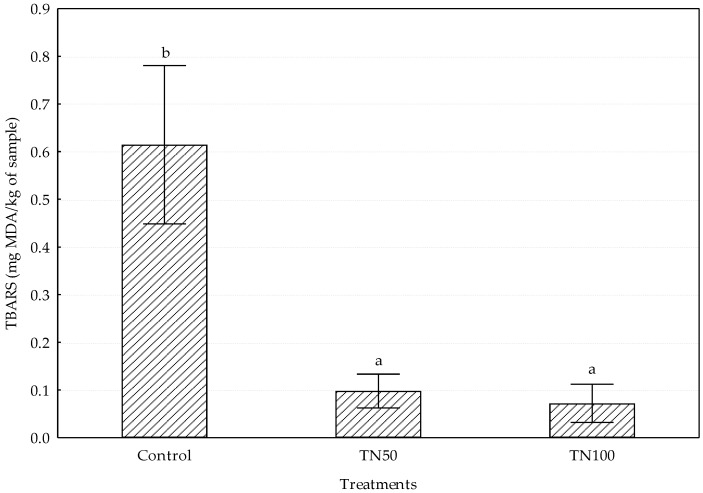
TBARS values of the different formulation burgers. ^a-b^ Different letter indicate statistically significant differences (*p* < 0.05).

TBARS were analyzed to assess the degree of lipid oxidation in the different samples, and the mean values of TBARS are shown in Figure 1. The Control formulation showed the highest TBARs value (0.61 ± 0.17 mg MDA/kg; *p* < 0.001) in comparison to TN50 (0.10 ± 0.04 mg MDA/kg) and TN100 (0.07 ± 0.04 mg MDA/kg) treatments. At this point, it should be noted that TBARS values were lower than the level at which consumers perceived rancidity.

### 3.2. Fatty Acid Profile of Beef Burgers

The fatty acids content of the different beef burger formulations is shown in Table 2. In this research, the presence and changes of the 41 fatty acids were studied. In the samples, 31 out of 41 fatty acids were detected, although only those that represented >0.1% are shown in Table 2. Despite this, all of them have been taken into account in the SFA, MUFA, PUFA, n-3, n-6 and nutritional indices. As expected, the replacement of beef fat by tiger nut oil emulsion had high influence in the content of all fatty acids detected. The major fatty acid in all samples was oleic acid (C18:1n-9; 36-48 g/100 g of total fatty acids), followed by palmitic acid (C16:0; 17–23 g/100 g of total fatty acids) and stearic acid (C18:0; 12–14 g/100 g of total fatty acids). The sum of these fatty acids represented about 74–78% of total fatty acids. The fourth most abundant fatty acid in the Control samples was 11t-C18:1 (TVA), while in the samples with partial or total substitution of beef fat was linoleic acid (C18:2n-6).

The addition of tiger nut oil emulsion as a substitute for beef fat was able to reduce SFA (*p* < 0.001) from 42.9 g/100 g in Control samples to 38.6 g/100 g in TN50 and 33.4 g/100 g in TN100. In fact, except for the content of C20:0, the other individual fatty acids showed a gradual decreased as fat animal was replaced.

In contrast, the content of MUFA increased (*p* < 0.001) from 50.2 g/100 g in Control samples to 53.2 and 55.1 g/100 g in TN50 and TN100, respectively. Obviously, this increase was directly related with the higher amounts of C18:1n-9 found in the samples that contain tiger nut oil emulsion. In this case, the other individual MUFA (except for C15:1n-5 and C20:1n-9) decreased as the proportion of tiger nut oil emulsion increased, especially the content of 11t-C18:1 that was reduced from 5.25 g/100 g in Control samples to 3.31 and 2.40 g/100 g in TN50 and TN100, respectively.

As occurs in MUFA, an increase was observed in total PUFA content (*p* < 0.001). To this regard, the contents of 9t,11t-C18:2 and 9c,11t-C18:2 (CLA) decreased as tiger nut oil emulsion was included in the formulation, while all individual n-6 PUFA and n-3 PUFA suffer a significant increased as the beef fat was replaced by tiger nut oil emulsion. It is especially worth mentioning the increase of C18:2n-6 in samples containing tiger nut oil (5.55 and 8.84 g/100 g in TN50 and TN100, respectively) in comparison with Control samples (4.38 g/100 g).

In order to verify how the addition of tiger nut oil affects the nutritional quality of hamburgers, the nutritional indices n-6/n-3, PUFA/SFA, AI, TI and h/H were calculated. An increase n-6/n-3, h/H, PUFA/SFA ratios and decrease in AI and TI were observed by replacing beef fat with tiger nut oil (Table 2).

The addition of tiger nut oil emulsion in beef burger significantly increased (*p* < 0.001, Table 2) the n-6/n-3 ratio in TN100 samples (from 5.6-6.1 in Control and TN50 samples to 8.97 in TN100) and PUFA/SFA ratio. In this case, a progressive increase was observed. Control samples showed the lowest PUFA/SFA values (0.16) and TN100 the highest values (0.34), while intermediate values were found in TN50 samples (0.21).

In contrast, the use of tiger nut oil emulsion as beef fat replacer decreased the atherogenic index (0.62 in Control vs. 0.48 and 0.35 in TN50 and TN100, respectively) and thrombogenic index (1.33 in Control vs. 1.10 and 0.88 in TN50 and TN100, respectively). Finally, the h/H ratio suffered a progressive increase as the tiger nut oil emulsion amount increased in the burger formulation, from values of 1.68 in Control samples to 2.25 in TN50 and 3.18 in TN100.

### 3.3. Sensory Analysis of Beef Burgers

**Figure 2:**
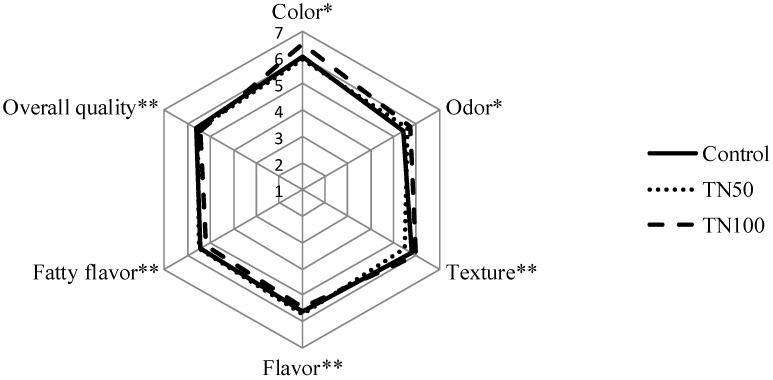
Acceptance test results of beef burger. * Raw burgers—color and odor. ** Cooked burgers—texture, flavor, fatty flavor and overall quality.

The results of the sensory acceptance test are shown in Figure 2. The color and odor attributes were assessed in raw burgers, while the other parameters (texture, flavor, fatty flavor and overall quality) were analyzed in cocked samples. The statistical analysis showed that the partial and total replacement of beef fat by tiger nut oil emulsion did not affect the sensory quality of burgers. In fact, all sensory parameters evaluated presented similar scores (*p* > 0.05).

**Figure 3:**
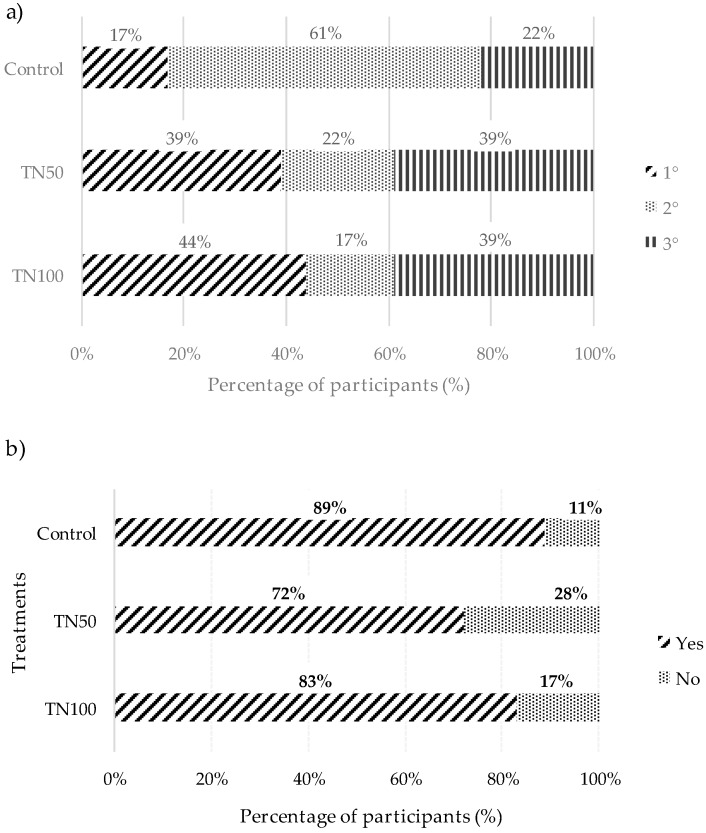
Results for the preference ordination (**a**) and purchase intentions (**b**) assays of beef burger.

Figure 3 indicates the results of consumer preference ordination (a) and purchase intentions (b) for different beef burgers treatments. The Control, TN50 and TN100 were the most preferred by 17, 39 and 44% of panelists, respectively (Figure 3a). However, non-significant differences were obtained from the preference test, which indicated similar preference among the three treatments. For purchase intention (Figure 3b), statistical analysis indicated that non-significant differences were obtained among treatments. Moreover, the percentage of positive answers was high for all treatment (89, 72 and 83% of consumers for Control, TN50 and TN100 treatments, respectively).

## 4. Discussion

### 4.1. Proximate Composition and Physicochemical Parameters of Beef Burgers

The proximate composition results showed a significant decrease of fat and protein, while moisture increased in the burgers with total replacement of beef fat by tiger nut oil emulsion (TN100). However, the replacement of 50% animal fat did not show differences in comparison with Control samples. Our results are in agreement with those reported by other authors in previous studies. To this regard, the pork back fat replacement by gelled emulsion containing sunflower oil resulted in a significant increase of moisture content [12], while the decrease of protein was also reported in recent studies with replacement of 20 to 100% of pork back fat by hydrogelled emulsion with chia and linseed oils in beef burger [3]. However, this reduction not only happens in burgers, but in other meat products. A significant protein decreased was reported in samples added with oil-in-konjac matrix (containing olive, linseed and fish oils) as a substitute (10% and 20%) of pork back fat in dry fermented sausages [37] and in liver pâté with partial substitution of pork fat (50% and 75% of substitution) by fish oil [38].

In agreement with the results obtained in the present study, several researches observed a significant decrease of fat content in burgers with animal fat replacement. In fact, both, the total replacement of animal fat by gelled emulsion with algae oil [4] and the partial replacement of pork back fat by a mixture of 10% back fat and 5% canola oil [14] reduced total fat content in the beef burgers. The partial replacement of pork back fat by microencapsulated fish oil also decreased the fat content in Frankfurter sausages [39] and dry-cured sausages [7].

The differences in proximate composition found in the present research between Control and TN100 samples are mainly due to the different composition between beef fat and tiger nut oil emulsion. As reported in the Materials and Methods section, the oil emulsion was composed by 56% of water and 37.3% of oil. Thus, it is expected that the animal replacement by oil emulsion result in a moisture increase and fat decrease. Additionally, no proteins were added to the emulsion, while the animal fat contains about 10% of proteins [3], which explain the diminution of protein content in TN100 in comparison with Control samples. It is also worth mentioning that all formulations of the present study can be considered as “high in protein” according to European Regulation [40] (at least 20% of the energy value of the food is provided by protein).

The content of ash increased as animal fat decreased in burger formulations. In contrast to our results, other authors did not find any differences in ash contents between the control formulation and treatments containing sesame oil oleogels as partial substitute (25% and 50%) of animal fat in beef burger [11]. This variation in ash content in the present study may be related to the addition of Prosella in the emulsion.

On the other hand, the TN100 samples presented the lowest content of carbohydrates, which suggested that it is probably due to the increase in moisture observed in TN100 samples. In contrast to our results, other authors did not find differences in carbohydrate content between control and modified burgers (mixture of pre-emulsified olive, corn and fish oil) [13]. The differences between both studies could be due to these authors employed corn (mainly composed of carbohydrates) and different proportions of water in the emulsion.

Finally, the low energy content observed in TN100 samples is related with the variations on proximate composition among TN100 and the other two batches. It is well known that fat is the most important component of calorie content [13]. Thus, a reduction in fat content (as occurs in TN100) provides a decrease in calorie content. Similar trends were reported in different meat products with animal fat substitution by other healthy oils [4,37].

However, pH values found in the present research were similar to those found in beef burgers by other authors [41,42]. Besides the significant differences observed among treatments, there is no clear trend and the values were similar.

Regarding color parameters, the beef fat replacement increased progressively L* and b* values, while a* values did not showed differences among batches. In agreement with our results, other studies in which oil emulsions replaced fat did not affect a* values in burgers [11,14]. The differences found in L* and b* were due to the replacement of beef fat by tiger nut oil emulsion, which have different compositions and consequently different tones of color. The oil globules diameter of the emulsions are smaller than the animal fat globules, which provides greater light reflection, thus increase L* values [43]. Tiger nut oil has a gold-yellow color [44]. The increase in b* may be justified because the tiger nut oil has a yellowish coloration while the beef fat has a whitish color (visual assessment). In agreement with the results found in the present research, different authors reported that b* values increased in burgers with animal fat replacement by olive and linseed oils [15] or by pre-emulsified olive, corn and fish oil [13]. Also was observed an increase in b* in raw hamburger with 80% and 100% of replacement of animal fat by hydrogelled emulsion with chia and linseed oils [3].

Cooking loss decreases in samples from total replacement of animal fat by tiger nut oil emulsion. This reduction may be due to the presence of Prosella in the emulsion, which acted as a barrier in liquid loss during cooking. Similar results were observed in beef burgers in which animal fat was replaced by vegetable oils resulted in a decrease in cooking loss [11,15].

Texture parameters were not influenced by animal fat replacement, despite the differences found in the proximate composition and cooking loss. In fact, only the springiness parameter increased by total replacement of beef fat by tiger nut oil emulsion. The hardness, cohesiveness, gumminess and chewiness were similar in all formulations. In contrast to our results, a hardness increase and springiness decrease was observed between the control beef burger treatment and the formulation containing 100% replacement of beef fat by canola and olive oil [2]. However, these authors also did not find significant difference for the cohesiveness, chewiness and gumminess parameters between treatments [2]. In dry-cured sausages, it was observed a decrease in hardness and increase in springiness and cohesiveness when 20% of animal fat were replaced by oil-in-konjac matrix [37], while another study reported an increase in hardness, gumminess and chewiness with 25, 50 and 75% of fat substitution by oil-in-konjac matrix [7]. In pâté, the fat replacement by oils resulted in a decrease in hardness and gumminess parameters [38,45]. Thus, it seems clear that the differences observed among the studies can be attributed to the different characteristics of emulsions used in meat products.

Regarding lipid oxidation, it is well known that highly UFA are more susceptible to the oxidative degradation than SFA [46]. With this in mind, it would be expected that the replacement of animal fat by tiger nut oil emulsion could promote oxidation in beef burgers. In fact, some researches in different meat products that employed fish oil (with high PUFA contents) as fat replacer resulted in a significant increase of TBARS and lipid oxidation-derived volatile compounds [7,38]. However, as could be seen in the results section, samples from TN50 and TN100 treatments showed very low TBARS values in comparison with Control. This demonstrates that tiger nut oil used had a very low degree of oxidation, probably lower than that of the beef fat. Additionally, oleic acid (the major fatty acid in chufa) is less susceptible to oxidative processes than PUFAs, which explain the differences between our results and those described in meat products reformulated with fish oils. In agreement with our findings, the total replacement of pork back fat by algae oil emulsion in beef burgers resulted in lower TBARS values [4]. The authors also argued that the low amount of oil used in the emulsion (1% algae oil), the presence of natural antioxidants in the oil, and the protection against oxidizing agents provided by the gelled emulsion (immobilized oil) could explain this outcome.

Despite the highest TBARS values found in Control burgers (0.61 mg MDA/kg) in the present research, all batches were kept below the limit of threshold for the oxidation acceptability. To this regard, different values ranging from 1 mg MDA/kg [47] to 2.5 mg MDA/kg [48] were reported in the literature as the threshold for sensorial perception of rancidity.

### 4.2. Fatty Acids of Beef Burgers

As expected, the fatty acids profile was highly affected by fat replacement. The most important impact was the reduction of SFA and the increase of MUFA and PUFA. Similarly, the animal substitution also influenced the fatty acid profile in other researches. About this, a reduction in SFA was also observed in burger with replacement of animal fat by vegetable oils [3,5]. Other studies that use olive oil as fat replacer also found a significant increase of MUFA, especially oleic acid, in modified burgers [13] and pâté [45]. Obviously, the studies with other meat products that use fish oils as fat substitutes resulted in an increase of total PUFA and especially long-chain n-3 fatty acids [7,38,39].

It is worth noting certain changes observed in some individual fatty acids. The most important variations were observed in the contents of C16:0, C18:0 and 11t-C18:1 that decreased significantly, while the contents of C18:1n-9, C18:2n-6 and C18:3n-3 increase with the inclusion tiger nut oil in burger formulation. Additionally, from nutritional point of view is important to highlight the progressive increase of long-chain n-3 fatty acids as eicosapentaenoic acid (C20:5n-3; EPA) and docosapentaenoic acid (C22:5n-3; DPA) in burgers with inclusion of tiger nut oil emulsion. These changes are directly related to the fatty acid composition of tiger nut oil. The oil used in the present study is characterized by their low content of SFA (Total SFA [21.0%], C16:0 [14%] and C18:0 [5.6%]) and high content of C18:1n-9 (67.2%) and C18:2n-6 (10.2%). In contrast, the content of TVA in the burgers decreased as the content of tiger nut oil increased in the formulation. It is well known that fat and meat of beef are rich in this fatty acid [49]. Thus, the incorporation of tiger nut, which do not have TVA, resulted in a dilution of this fatty acid in the reformulated burgers. As general conclusion, it seems that the fatty acids profile of the burgers perfectly reflects the fatty acids of the fat/oil source used in their formulation.

On the other hand, except for n-6/n-3 ratio that increased as increase the beef fat replacement, the inclusion of tiger nut oil emulsion improved progressively the other nutritional indices. All treatments exceed the recommendation for n-6/n-3 ratio, which must be less than 4 [50]. As indicated above, the tiger nut oil is mainly composed of oleic acid, a MUFA, and has low content of omega-3 fatty acids (Table 2). Therefore, the replacement of beef fat by tiger nut oil emulsion in burger was not able to modify both omega-6 and omega-3 fatty acids. It is also relevant mentioning that the outcomes derived from the n-6/n-3 ratio should not be considered alone. This concern is supported by the physiological impacts of each PUFA has on the organism, which should not be evaluated by a single ratio [51]. The PUFA/SFA ratio increase as tiger nut was included in the formulation and TN100 samples presented similar values (0.34) to those recommended (about 0.4) [52].

Regarding the other nutritional indices, it is possible to observe in Table 2 a decrease in AI and TI and increase in h/H (*p* < 0.001) with the addition of tiger nut oil emulsion in burger. The recommendations for a healthier diet is that AI and TI should be as low as possible [34], while the h/H ratio should be high. Similar to the present study, other authors observed a reduction in AI and TI in burgers containing vegetable oil emulsion as a fat substitute [3,5]. Not only burgers, but in other meat products a decrease in AI and TI and increasing the h/H ratio with the substitution of animal fat by healthy oils was observed [38,39,45]. Finally, all treatments (Control, TN50 and TN100) can be claimed as “low-SFA” [40], once the Control treatment was reformulated with low amount of beef fat (5 g/100 g).

### 4.3. Sensory Analysis of Beef Burgers

As commented above, the beef fat substitution by tiger nut oil emulsion had no influence in the sensory parameters acceptability (Figure 2). It is possible to affirm that all the formulations were considered “well-accepted”, receiving scores of “5 = liked slightly” and “7 = liked much”. Similar to the present study, it was reported that the use of olive and flaxseed oil’s mixture as a partial substitute for animal fat did not affect the burgers sensory parameters [15]. Other study also verified that the 50% replacement of animal fat by sesame oil oleogels was considered acceptable by consumers [11], while the substitution of up to 60% of animal fat by hydrogelled emulsion with chia and linseed oils presented a higher acceptability compared to the control burgers (containing 100% pork back fat) [3].

## 5. Conclusions

The total replacement of beef fat by tiger nut oil emulsion in beef burger resulted in a healthier meat product, with low total fat and SFA contents and rich in UFA. The main MUFA found in the composition was oleic acid, which can provide health benefits. In addition, the nutritional indices (except for n-6/n-3 ratio) showed that the inclusion of tiger nut oils improve the nutritional quality of burgers. Moreover, the total replacement of beef fat was considered acceptable by consumers. Thus, as a general conclusion the use of tiger nut oil emulsion in the burger formulation instead beef fat allows develop a healthier product with high consumer acceptability.

## Figures and Tables

**Table 1:** Proximate composition and physicochemical results of beef burgers.

	Treatments			SEM	Sig.
	Control	TN50	TN100		
**Proximate composition (g/100 g)**					
Moisture	73.19 ^a^	73.11 ^a^	75.37 ^b^	0.231	***
Fat	4.42 ^b^	4.34 ^b^	3.01 ^a^	0.166	***
Protein	19.21 ^b^	19.12 ^b^	18.75 ^a^	0.057	***
Ash	2.09 ^a^	2.23 ^b^	2.29 ^b^	0.025	**
Carbohydrates	1.09 ^b^	1.20 ^b^	0.58 ^a^	0.090	**
Energy content (kJ/100 g)	508.5 ^b^	506.1 ^b^	439.9 ^a^	7.268	***
pH	5.58 ^b^	5.48 ^a^	5.76 ^c^	0.028	***
**Color parameters**					
L *	44.13 ^a^	45.02 ^a,b^	46.94 ^b^	0.468	*
a *	20.86	21.17	21.83	0.370	ns
b *	19.19 ^a^	19.89 ^a,b^	20.88 ^b^	0.284	*
Cooking loss (%)	22.34 ^b^	24.32 ^b^	13.27 ^a^	1.047	***
**Texture parameters**					
Hardness (N)	174.1	185.5	177.0	2.606	ns
Springiness (mm)	0.80 ^a^	0.80 ^a^	0.85 ^b^	0.005	***
Cohesiveness	0.62	0.62	0.62	0.002	ns
Gumminess (N)	108.7	115.8	109.3	1.632	ns
Chewiness (N·mm)	87.68	93.34	92.69	1.318	ns

^a–c^ Different letters on the same row (same parameter) indicate significant differences (*p* < 0.05; Tukey test). SEM: Standard error of mean. Sig.: significance; ns: not significant. * *p* < 0.05; ** *p* < 0.01; *** *p* < 0.001. Control—content 100% beef fat; TN50—50% of beef fat replaced by tiger nut oil; TN100—100% of beef fat replaced by tiger nut oil.

**Table 2:** Fatty acid profile of beef burgers.

	Treatments			SEM	Sig.
	Control	TN50	TN100		
**Fatty acids (g/100 g of total FA)**					
C14:0	2.78 ^c^	2.06 ^b^	1.32 ^a^	0.130	***
C15:0	0.51 ^c^	0.38 ^b^	0.28 ^a^	0.020	***
C16:0	23.86 ^c^	21.18 ^b^	17.74 ^a^	0.535	***
C17:0	1.31 ^c^	1.06 ^b^	0.71 ^a^	0.052	***
C18:0	14.16 ^c^	13.38 ^b^	12.45 ^a^	0.180	***
C20:0	0.11 ^a^	0.24 ^b^	0.52 ^c^	0.035	***
SFA	42.94 ^c^	38.57 ^b^	33.41 ^a^	0.830	***
C14:1n-5	0.59 ^c^	0.38 ^b^	0.17 ^a^	0.036	***
C15:1n-5	0.49 ^a^	0.55 ^b^	0.69 ^c^	0.019	***
C16:1n-7	3.39 ^c^	2.64 ^b^	1.19 ^a^	0.192	***
C17:1n-7	1.03 ^c^	0.82 ^b^	0.36 ^a^	0.059	***
9t-C18:1	0.73 ^b^	0.58 ^a^	0.52 ^a^	0.023	***
11t-C18:1	5.25 ^c^	3.31 ^b^	2.40 ^a^	0.252	***
C18:1n-9	36.87 ^a^	43.25 ^b^	48.40 ^c^	0.996	***
C18:1n-7	1.60 ^c^	1.45 ^b^	1.13 ^a^	0.041	***
C20:1n-9	0.18 ^a^	0.20 ^b^	0.20 ^b^	0.003	**
MUFA	50.16 ^a^	53.21 ^b^	55.11 ^c^	0.446	***
9t,11t-C18:2	0.35 ^c^	0.30 ^b^	0.09 ^a^	0.024	***
9c,11t-C18:2 (CLA)	0.37 ^b^	0.31 ^a^	0.28 ^a^	0.008	***
C18:2n-6	4.38 ^a^	5.55 ^b^	8.84 ^c^	0.397	***
C20:3n-6	0.19 ^a^	0.20 ^a,b^	0.21 ^b^	0.004	*
C20:4n-6	0.62 ^a^	0.74 ^b^	0.89 ^c^	0.027	***
n-6	5.24 ^a^	6.54 ^b^	9.99 ^c^	0.423	***
C18:3n-3	0.43 ^a^	0.49 ^b^	0.50 ^b^	0.010	**
C20:5n-3	0.15 ^a^	0.18 ^b^	0.19 ^b^	0.005	***
C22:5n-3	0.32 ^a^	0.34 ^a,b^	0.37 ^b^	0.009	*
n-3	0.94 ^a^	1.08 ^b^	1.12 ^b^	0.023	***
PUFA	6.90 ^a^	8.22 ^b^	11.48 ^c^	0.408	***
**Nutritional indices**					
n-6/n-3	5.60 ^a^	6.08 ^a^	8.97^b^	0.325	***
PUFA/SFA	0.16 ^a^	0.21 ^b^	0.34^c^	0.016	***
AI	0.62 ^c^	0.48 ^b^	0.35^a^	0.024	***
TI	1.33 ^c^	1.10 ^b^	0.88^a^	0.040	***
h/H	1.68 ^a^	2.25 ^b^	3.18^c^	0.131	***

^a–c^ Different letters on the same row (same parameter) indicate significant differences (*p* < 0.05; Tukey test). SEM: Standard error of mean. Sig.: significance; * *p* < 0.05; ** *p* < 0.01; *** *p* < 0.001. Control—content 100% beef fat; TN50—50% of beef fat replaced by tiger nut oil; TN100—100% of beef fat replaced by tiger nut oil. SFA: saturated fatty acids. MUFA: monounsaturated fatty acids. PUFA: polyunsaturated fatty acids. n-3: omega-3; n-6: omega-6; AI: Atherogenic index. TI: Thrombogenic index. h/H: hypo/hypercholesterolemic fatty acids ratio.
